# Supplementary material for: NMFClustering: Accessible NMF-based clustering utilizing GPU acceleration
Source: bioRxiv. 2023 Jun 27:2023.06.16.545370. Preprint. [Version 2] doi: 10.1101/2023.06.16.545370 (PMC10312797; doi:10.1101/2023.06.16.545370)
Supplement: Supplement 1 [file NIHPP2023.06.16.545370v2-supplement-1.pdf]

bioRxiv preprint doi: https://doi.org/10.1101/2023.06.16.545370; this version posted June 27, 2023. The copyright holder for this preprint (which was not certified by peer review) is the author/funder, who has granted bioRxiv a license to display the preprint in perpetuity. It is made available under aCC-BY 4.0 International license.

# Supplemental Data

## NMFClustering User Interface

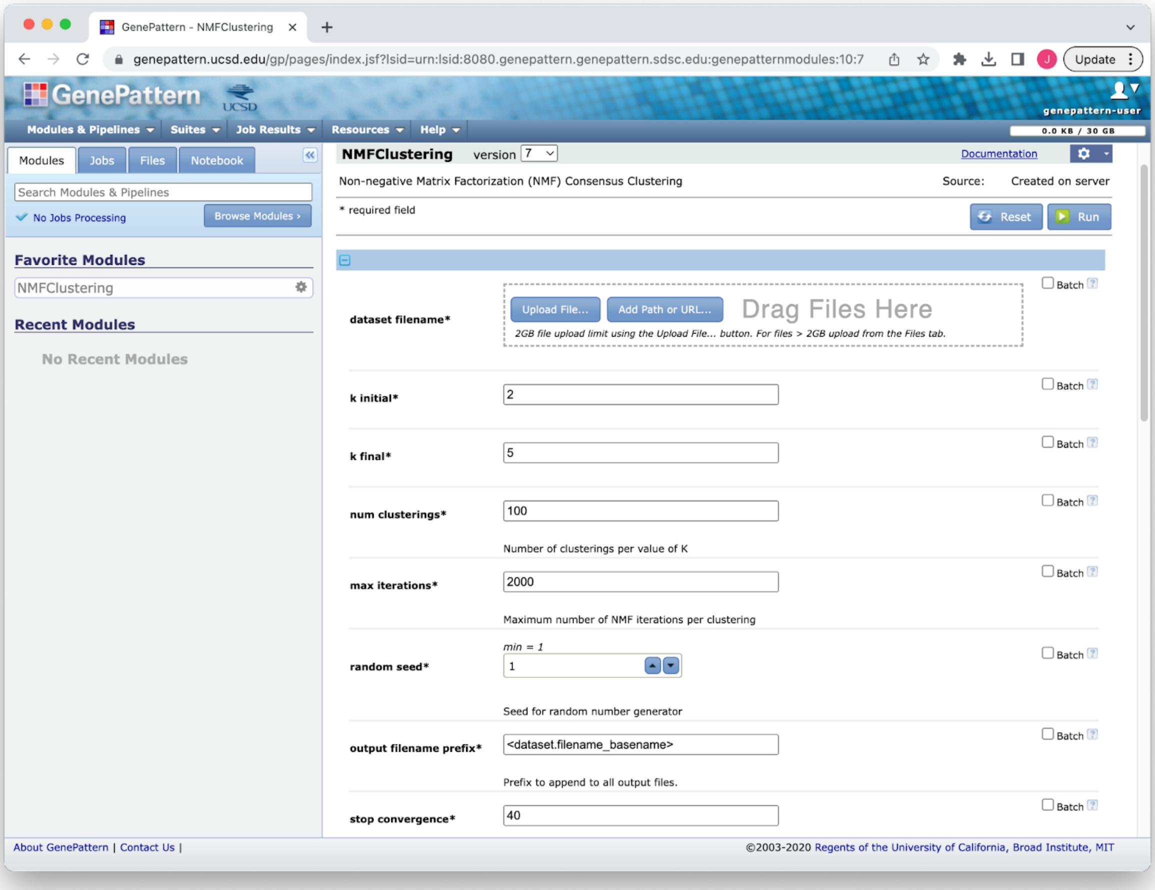


**Figure S1**, NMFClustering interface in GenePattern showing a subset of the available parameters

bioRxiv preprint doi: https://doi.org/10.1101/2023.06.16.545370; this version posted June 27, 2023. The copyright holder for this preprint (which was not certified by peer review) is the author/funder, who has granted bioRxiv a license to display the preprint in perpetuity. It is made available under aCC-BY 4.0 International license.

## Example Consensus Matrix plot

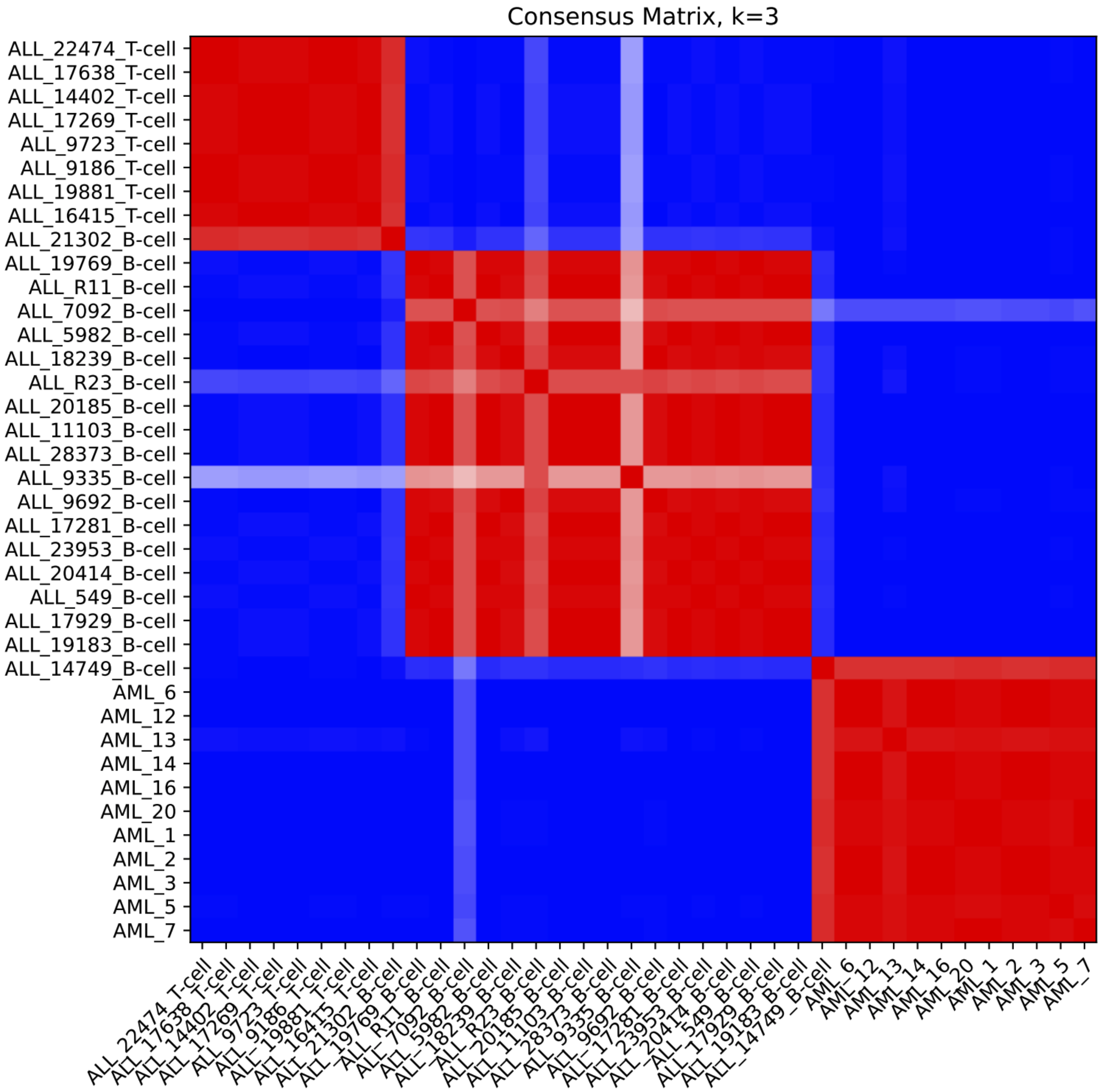


Figure S2, Example consensus matrix plot for k=3 using ALL_AML_data.gct dataset from the sample test data folder (below). Compare to figure 4, panel a in Brunet et al.

## Sample Test Data

Test datasets are available at
https://datasets.genepattern.org/?prefix=data/test_data/NMFClustering/
